# Mortality Attributable to Seasonal Influenza A and B Infections in Thailand, 2005–2009: A Longitudinal Study

**DOI:** 10.1093/aje/kwu360

**Published:** 2015-04-20

**Authors:** Ben S. Cooper, Surachai Kotirum, Wantanee Kulpeng, Naiyana Praditsitthikorn, Malinee Chittaganpitch, Direk Limmathurotsakul, Nicholas P. J. Day, Richard Coker, Yot Teerawattananon, Aronrag Meeyai

**Keywords:** Bayesian regression, burden, developing country, influenza, middle-income country, mortality, seasonal variation, tropics

## Abstract

Influenza epidemiology differs substantially in tropical and temperate zones, but estimates of seasonal influenza mortality in developing countries in the tropics are lacking. We aimed to quantify mortality due to seasonal influenza in Thailand, a tropical middle-income country. Time series of polymerase chain reaction–confirmed influenza infections between 2005 and 2009 were constructed from a sentinel surveillance network. These were combined with influenza-like illness data to derive measures of influenza activity and relationships to mortality by using a Bayesian regression framework. We estimated 6.1 (95% credible interval: 0.5, 12.4) annual deaths per 100,000 population attributable to influenza A and B, predominantly in those aged ≥60 years, with the largest contribution from influenza A(H1N1) in 3 out of 4 years. For A(H3N2), the relationship between influenza activity and mortality varied over time. Influenza was associated with increases in deaths classified as resulting from respiratory disease (posterior probability of positive association, 99.8%), cancer (98.6%), renal disease (98.0%), and liver disease (99.2%). No association with circulatory disease mortality was found. Seasonal influenza infections are associated with substantial mortality in Thailand, but evidence for the strong relationship between influenza activity and circulatory disease mortality reported in temperate countries is lacking.

The World Health Organization asserts that seasonal influenza results in 250,000–500,000 deaths annually in industrialized countries (1). Estimates are lacking from low- and middle-income countries but might differ substantially because of reduced health-care resources, differences in influenza transmission dynamics, poorer nutrition, differences in chronic illnesses, and lower levels of vaccine coverage against influenza and interacting pathogens, such as *Streptococcus pneumoniae* (2, 3). Differences in the pattern of person-to-person contacts that spread influenza may also lead to different patterns of epidemic spread that could potentially affect influenza-related mortality. Such differences in contact patterns may relate to the degree of urbanization and the demographic structure of the population. Finally, many developing countries are in tropical or subtropical zones, where influenza seasonality and strain diversity can differ substantially from those of temperate regions (3). Very little is currently known about mortality due to seasonal influenza in the tropics with the exception of one very highly developed and fully urbanized population, Singapore (4, 5).

Although routine influenza vaccination programs have previously been limited to higher-income settings, there is increasing interest in expanding such programs in developing countries. Estimating influenza-related mortality in such settings is essential for quantifying the likely impact of such vaccination programs (1). However, a major challenge in estimating seasonal influenza mortality is that symptoms are nonspecific, and few patients are tested for active influenza infection. It is also evident that, for most deaths where influenza is likely to have played a causal role, no mention of influenza is given as the cause of death (2, 4–7).

To overcome these limitations, approaches have been developed to estimate influenza-related mortality by using routine surveillance data (2, 4–12). Most aim to separate mortality time series into a predictable component of temporal variation (annual periodicity and long-term trends) and, added to this, a variable component. Mortality associated with influenza is expected to be largely explained by the latter. A seminal approach, the Serfling model (9), used a sine wave to model the regular seasonal component of variation and a polynomial in time to account for long-term trends. Mortality exceeding this baseline model in “epidemic months” is termed “excess mortality.” Much of this excess, it is argued, is caused by influenza.

Because of several limitations of this model (likely to be particularly severe in tropical and subtropical settings), recent work uses more flexible functional forms (splines) for modeling underlying seasonality and regression models to relate mortality to influenza activity (2, 4, 6, 12). We adopt a similar approach to estimate mortality due to seasonal influenza in Thailand, adjusting for nonlinear associations of mortality with meteorological data. We extend previous approaches by allowing for year-on-year variation in the relationship between subtype-specific influenza activity and mortality.

We use data collected prospectively by the national influenza surveillance system in Thailand. This combines epidemiologic and virological data and was set up by the Thai National Institute of Health at the Ministry of Public Health in 2004 in collaboration with the US Centers for Disease Control and Prevention (13).

## METHODS

### Data sources

Weekly deaths in Thailand for 2005–2009 were obtained from the Ministry of Public Health and included both all-cause mortality and mortality with codes from the *International Classification of Diseases, Tenth Revision* (ICD-10), for respiratory disease (J00–J99), circulatory disease (I00–I99), cancer (C00–C97), diabetes (E10–E14), renal disease (N00–N07, N17–N19, N25–N27), and liver disease (K70, K73, K74). These have previously been reported to be associated with influenza. We also included 2 control categories that have not been reported to be associated with influenza: septicemia (A40, A41) and unintentional injuries (V01–X59, Y85, Y86) (2).

Influenza data consisted of reports of weekly numbers of patients seeking medical attention with influenza-like illness throughout Thailand and weekly laboratory-confirmed influenza cases from April 2005 to March 2009. These dates were chosen because the influenza surveillance system was not fully operational until 2005 and, following March 2009, health care–seeking behavior for influenza-like illness changed in response to pandemic influenza (13). Visual inspection indicated anomalies in the data in weeks 52, 53, and 1 of each year, and these were excluded from the analysis. Laboratory confirmation data included the number of weekly tests from patients with influenza-like illness and the number positive for influenza A(H3N2), A(H1N1), and B (13). Three meteorological measurements were used: maximum temperature, relative humidity, and rainfall. These were averaged over daily readings from Bangkok obtained from the Thai Meteorological Department. Six out of over 3,000 observations were missing or represented coding errors and were replaced with the last observation carried forward.

### Statistical analysis

We defined an influenza activity measure, *B_j_*_,*t*_, as the product of the proportion of laboratory tests positive for influenza type *j* in week *t* and the number of patients with influenza-like illness in week *t*. This is similar to a previously described incidence proxy (2). The number of patients with influenza-like illness due to noninfluenza causes may vary over time, as may the probability that laboratory confirmation for influenza is sought in a patient with influenza-like illness. The activity measure, *B_j_*_,*t*_, will not be affected by such variation and will be proportional to the number of true cases with influenza type *j* in week *t*, provided that the probability that a patient with an influenza type *j* infection seeks medical attention for influenza-like illness and test sensitivity do not vary with time (refer to the Web Appendix, available at http://aje.oxfordjournals.org/).

We estimated mortality due to influenza using regression models that expressed weekly deaths as the sum of a regular seasonal component of variation, a long-term trend, a contribution from each of the influenza types, a component related to meteorological conditions, and an error term. We used separate models for all-cause deaths, deaths in 3 different age groups (≤17, 18–59, ≥60 years), and deaths for specific groupings of ICD-10 codes. Seasonal variation was accounted for by using periodic penalized B-splines (P-splines) (14, 15).

Models considered were of the form

(1) $$\begin{array}{rl} D_{t} & =S_{t}+L_{t}+\sum_{\;j=1\ldots 3}\beta_{j}\times (\;f\times B_{\;j,t-1}+(1-f)\\ & \;\times B_{\;j,t-2})+W_{t}+\epsilon_{t}, \end{array}$$

where *D_t_* represents deaths in week *t*, *S_t_* corresponds to the week *t* contribution from the periodic seasonal term, *L_t_* corresponds to the week *t* contribution from the long-term trend term, the β*_j_* terms estimate the contribution to mortality from the 3 influenza types, *W_t_* corresponds to the week *t* adjustment for other covariates, and ϵ*_t_* is the residual. We followed previous work in assuming that deaths caused by influenza will lag influenza cases by 1–2 weeks; the *f* term represents the proportional contribution to deaths of influenza cases 1 week previously (2).

Regression models were developed by using a 2-stage approach where we first performed an exploratory analysis to select the best models and then estimated mortality using the selected model. In an initial exploratory stage, we compared 12 different generalized additive models, accounting for seasonal variation using P-splines, comparing approaches using low-order polynomials and P-splines for modeling the long-term trends, and comparing Gaussian models with Poisson models (with an identity link function). We used the Akaike Information Criterion to assess model fit (16). This showed that Gaussian models where long-term trends were modeled by either a P-spline or a quadratic function of the week number gave similar fits (Web Table 1). We used the latter assumption in the second stage, in which we implemented models within a Bayesian framework and estimated parameters using a Markov chain Monte Carlo approach, because it gave greatly improved mixing of the Markov chain (Web Figure 1). This enabled us to estimate the type-specific delay, *f*, from reported influenza to death*,* account for temporal autocorrelation between observations by using a second-order random-walk prior, and consider 3 different assumptions about how the coefficients in the regression model relating influenza activity to mortality varied by year. For cause-specific mortality, we report the posterior probability of positive association with influenza (i.e., the posterior probability that influenza-associated mortality is greater than zero). We considered 3 different approaches to adjusting for meteorological variables (no adjustment; adjustment assuming linear relationships; and a semiparametric regression approach with P-splines to allow for nonlinear relationships between meteorological covariates and mortality). Finally, when we found evidence of heteroskedasticity, we extended the best-fitting model by allowing the variance to follow a first-order autoregressive conditional heteroskedastic process (17).

Mortality in weeks excluded from the model fitting was estimated by linear interpolation based on estimated mortality in weeks before and after excluded data. The assumed population size of each age group in each year was derived from census data (http://web.nso.go.th/en/census/poph/cen_poph.htm) and World Bank population estimates (http://databank.worldbank.org/). Full-model details are given in the Web Appendix.

Analysis was performed using R, version 2.13.0 (R Foundation for Statistical Computing, Vienna, Austria), with the package *mgcv* for initial model exploration and WinBUGS, version 1.4 (freeware), for the Bayesian analysis (18–20).

## RESULTS

**Figure 1.**
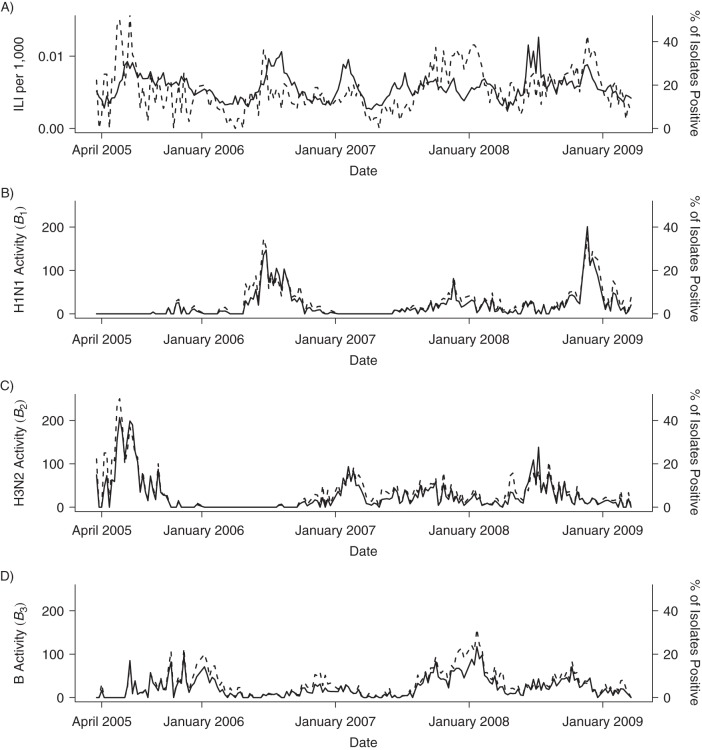
Time series of observed influenza-like illness (ILI) and influenza activity measures (*B*_1_, *B*_2_, *B*_3_) in Thailand, 2005–2009. Influenza activity measures represent the product of influenza-like illness and the percentage of tested isolates positive for a given type in each week. Broken lines show the proportion of tested isolates positive for influenza (A) and positive for specific influenza types (B–D).

**Figure 2.**
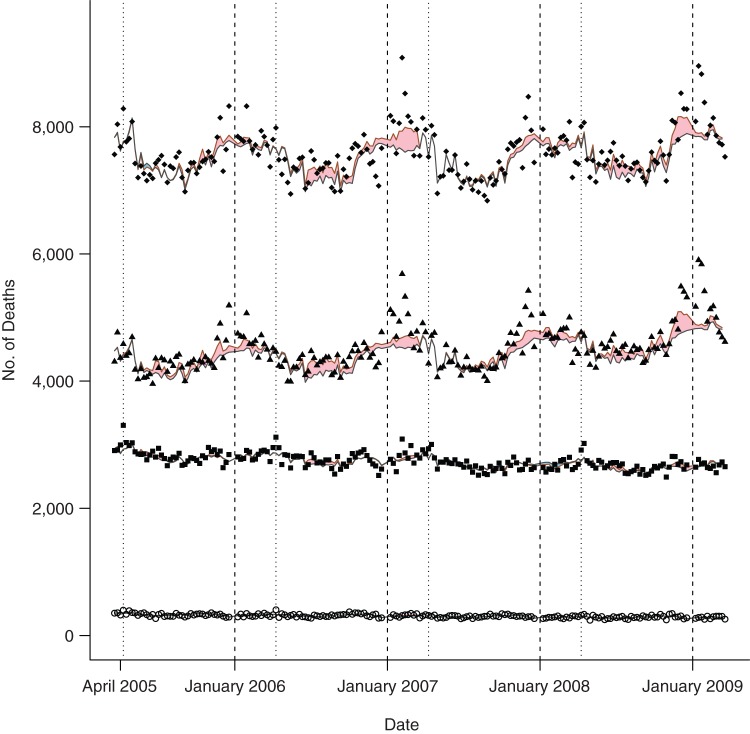
Observed and expected deaths by age group in Thailand, 2005–2009. The figure shows recorded weekly deaths in those aged ≤17 years (open circles), 18–59 years (squares), ≥60 years (triangles), all ages (diamonds), and the expected number of deaths predicted by the full model (red lines) and expected number of deaths excluding those due to influenza (black lines). Shaded areas indicate excess deaths attributed to influenza (pink if positive, blue if negative). Vertical dashed lines indicate the first week of the calendar year, and vertical dotted lines mark the Thai New Year (April 13–15).

**Figure 3.**
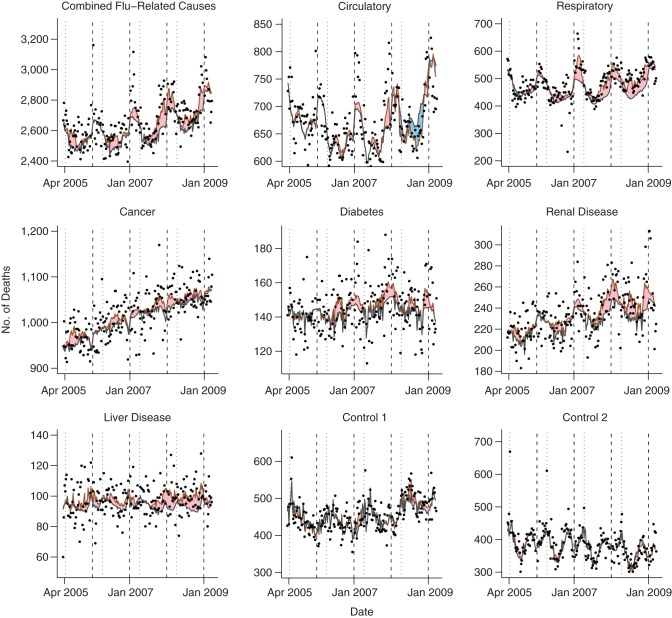
Observed and expected deaths attributed to causes commonly associated with influenza and 2 control causes in Thailand, 2005–2009. Shaded areas indicate excess deaths attributed to influenza (pink if positive, blue if negative). Vertical dashed lines indicate the first week of the calendar year, and vertical dotted lines mark the Thai New Year (April 13–15). Apr, April; Jan, January.

Although influenza B was endemic throughout the entire period, there were extended periods when 1 of the 2 influenza A subtypes was absent (Figure 1). No clear seasonal pattern was evident for any influenza type. All-cause mortality data, in contrast, showed a clear seasonal pattern, with peaks close to the end of each calendar year (Figure 2). This seasonal variation was not apparent in those aged <60 years. Combined mortality with classifications previously associated with influenza was elevated close to the end of each calendar year, reflecting annual peaks in mortality from circulatory and respiratory disease (Figure 3). There was also an increasing trend in mortality attributed to influenza-related causes, reflecting increasing mortality from cancer, diabetes, and renal disease. The 2 control causes of mortality also showed seasonal patterns: Septicemia deaths showed troughs close to the end of each calendar year but peaks shortly after the Thai New Year (April 13–15), and unintentional injury deaths showed large peaks during the Thai New Year.

In the models relating influenza activity to all-cause mortality, background seasonal mortality (not explained by influenza) peaked near the start of the calendar year and was at a minimum at about week 30 (Web Figure 2). Adjustment for meteorological data substantially improved model fit (Web Table 2). This showed that, superimposed on the regular seasonal variation, all-cause mortality decreased at high levels of relative humidity and increased at high temperatures (Web Figure 3). There was no evidence of any association with rainfall. Allowing for year-to-year variation in the association between influenza activity and mortality also improved model fit. The best model overall accounted for both a nonlinear association between meteorological data and mortality and for year-to-year variation in the association between influenza activity and mortality. We report results for this model allowing for time-dependent variance below. Results obtained by using alternative models are shown in Web Tables 3–9.

The overall posterior mean of 6.1 deaths per 100,000 population per year corresponds to approximately 4,000 annual deaths due to influenza in Thailand (Table 1).

**Table 1.** Estimated Number of Influenza-Related Deaths per 100,000 Population, by Age Group, Thailand, 2005–2009^a^

Age Group, years	Influenza A(H1N1)		Influenza A(H3N2)		Influenza B		Total Influenza A		Total Influenza A and B	
	Mean	95% CrI	Mean	95% CrI	Mean	95% CrI	Mean	95% CrI	Mean	95% CrI
All ages	2.6	0.8, 5.1	1.6	−1.7, 5.4	1.9	−3.5, 6.9	4.2	−0.2, 9.2	6.1	0.5, 12.4
≤17	0.0	−0.8, 0.8	0.6	−0.4, 1.6	−0.4	−2.0, 1.3	0.6	−0.8, 2.1	0.1	−0.4, 0.6
18–59	1.4	0.3, 2.7	1.4	−0.4, 3.1	−1.7	−4.0, 0.6	3.0	0.5, 5.6	1.1	−1.7, 4.0
≥60	28.1	11.1, 45.9	13.0	−7.5, 34.5	26.9	−7.7, 62.2	41.0	13.7, 69.2	68.0	27.2, 108.1

Abbreviation: CrI, credible interval.

^a^ Estimates were obtained by using the model adjusting for meteorological data and allowing for annual variation in the association between type-specific influenza activity measures and mortality using a random-effects model.

There was considerable variation between the 4 years in both total mortality due to influenza A and the relative importance of H1N1 and H3N2 (Table 2).

**Table 2.** Estimated Number of Influenza-Related Deaths per 100,000 Population, by Year, Thailand, 2005–2009^a^

Year	Influenza A(H1N1)		Influenza A(H3N2)		Influenza B		Total Influenza A		Total Influenza A and B	
	Mean	95% CrI	Mean	95% CrI	Mean	95% CrI	Mean	95% CrI	Mean	95% CrI
2005–2006	0.4	−0.1, 0.9	−0.5	−6.1, 5.1	3.3	−2.1, 10.6	−0.1	−5.7, 5.6	3.1	−4.6, 11.9
2006–2007	4.1	0.9, 7.5	3.6	−0.9, 13.2	1.2	−2.9, 6.7	7.7	1.6, 17.8	9.0	2.2, 20.1
2007–2008	2.2	−0.2, 5.8	−0.4	−6.9, 5.6	1.5	−7.2, 8.6	1.8	−5.3, 8.7	3.2	−6.5, 12.3
2008–2009	4.1	0.7, 8.4	3.7	−1.6, 9.4	1.4	−9.0, 8.8	7.8	1.1, 15.5	9.2	−2.3, 19.3

Abbreviation: CrI, credible interval.

^a^ Estimates were obtained by using the model adjusting for meteorological data and allowing for annual variation in the association between type-specific influenza activity measures and mortality using a random-effects model.

H1N1 was associated with only a small increase in mortality in 2005–2006 when H1N1 activity was low but with 2–4 deaths per 100,000 population over the next 3 years. Annual mortality associated with H3N2 was even more variable: In 2005–2006, despite evidence of a large H3N2 epidemic, there was no association with increased mortality, while annual associated mortality subsequently ranged between 0 and 4 deaths per 100,000.

Alternative models gave broadly similar results, although constant-variance models consistently found no evidence that influenza B made any contribution to mortality (Web Tables 3–9). Models that accounted for annual variation in the association between influenza activity and mortality all estimated a larger number of deaths attributed to influenza A than models that did not. In all cases, adjusting for this annual variation led to improved model fit. There was evidence that the relationship between influenza activity and mortality varied over the 4 years for A(H3N2) but not for A(H1N1) (Web Figure 4). In particular, each unit of activity with A(H3N2) in 2006–2007 was associated with a far greater mortality risk than in the other 3 years (Web Table 10). There was no strong correlation between total annual deaths attributed to the 3 different influenza types and at most weak correlation between their year-specific regression coefficients (Web Figure 5).

Almost all influenza-related deaths were estimated to occur in those aged ≥60 years, with an estimated 68 influenza-related deaths annually per 100,000 people in this age group—about 2% of the age group's total mortality rate (Table 1). There was also evidence of an association between influenza A and increased mortality in those aged 18–59 years, with about 3 deaths per 100,000 population per year or 0.3% of the total mortality rate. In those aged <18 years, we estimated less than 1 death per 100,000 per year due to influenza, 0.1% of the total mortality rate. In these age group–specific analyses, influenza B was estimated to account for 40% of the influenza-related mortality in those ≥60 years. There was no evidence of mortality associated with influenza B in other age groups.

Analysis of cause-specific mortality found evidence that influenza was associated with deaths attributed to respiratory disease (probability of positive association, 99.8%, based on adjusted model), cancer (98.6%), diabetes (99.3%), renal disease (98.0%), and liver disease (99.2%). The highest mortality attributed to influenza (1.6 per 100,000 per year) was seen in respiratory disease deaths; about 4% of deaths in this category were attributed to influenza (Table 3). Again, these deaths were mostly in those aged ≥60 years, and in each year apart from 2005–2006, influenza A was estimated to play a dominant role (Table 4). For the 2 control causes of death, we found little evidence of any association with influenza (Table 3; Figure 3). Posterior probabilities for a positive association between influenza and excess mortality were 87.2% for control 1 and 56.5% for control 2 (all prior probabilities were 50%).

**Table 3.** Cause-Specific Mortality^a^ Attributable to Influenza for Major ICD-10 Groupings, Thailand, 2005–2009^b^

Recorded Cause of Death	Mean Annual Mortality per 100,000 Population	Mortality per 100,000 Due to Influenza		Cause-Related Deaths Attributed to Influenza, %		*f*_A_^c^		*f*_B_^c^	
		Mean	95% CrI	Mean	95% CrI	Mean	95% CrI	Mean	95% CrI
Circulatory	51.5	0.0	−0.8, 0.8	−0.1	−1.6, 1.5	0.43	0.07, 0.85	0.51	0.16, 0.87
Respiratory	36.8	1.6	0.5, 2.5	4.1	1.4, 7.0	0.49	0.20, 0.80	0.34	0.01, 0.92
Cancer	77.7	0.8	0.0, 1.7	1.2	0.0, 2.2	0.54	0.04, 0.98	0.37	0.01, 0.93
Diabetes	11.0	0.4	0.1, 0.7	3.8	1.1, 6.6	0.44	0.03, 0.94	0.49	0.03, 0.96
Renal disease	17.6	0.4	0.0, 0.8	2.3	0.1, 4.5	0.51	0.05, 0.96	0.46	0.03, 0.96
Liver disease	7.4	0.3	0.1, 0.6	4.4	0.9, 7.7	0.51	0.03, 0.97	0.86	0.46, 1.00
Above causes combined	202.5	3.7	1.4, 6.0	1.8	0.7, 3.0	0.48	0.08, 0.89	0.48	0.02, 0.97
Control 1: septicemia	34.6	0.2	−0.4, 0.8	0.7	−1.2, 2.6	0.58	0.14, 0.96	0.48	0.03, 0.97
Control 2: unintentional injuries	29.3	0.0	−0.8, 0.8	0.2	−2.7, 3.0	0.58	0.06, 0.98	0.52	0.03, 0.97

Abbreviations: CrI, credible interval; ICD-10, *International Classification of Diseases, Tenth Revision*.

^a^ “Mortality” was defined as number of deaths.

^b^ Estimates were obtained by using the model adjusting for meteorological data and allowing for annual variation in the association between type-specific influenza activity measures and mortality using a random-effects model.

^c^ Refer to equation 1.

**Table 4.** Estimated Number of Influenza-Related Deaths Classified as Due to Respiratory Causes per 100,000 Population, by Year and Age Group, Thailand, 2005–2009^a^

Age Group and Year	Influenza A(H1N1)		Influenza A(H3N2)		Influenza B		Total Influenza A and B	
	Mean	95% CrI	Mean	95% CrI	Mean	95% CrI	Mean	95% CrI
Age ≥60 years								
All years	4.1	1.3, 7.0	5.7	2.4, 9.0	1.8	−2.2, 6.2	11.6	6.1, 17.0
2005–2006	0.2	−1.8, 1.6	−1.8	−6.6, 3.4	2.8	−2.2, 8.3	1.1	−5.5, 8.4
2006–2007	2.3	−1.9, 6.5	8.9	4.6, 13.4	0.1	−6.8, 3.8	11.3	3.8, 18.1
2007–2008	7.0	0.9, 15.4	5.7	−1.1, 12.9	1.6	−5.6, 8.4	14.3	5.8, 23.0
2008–2009	6.2	2.0, 10.0	9.5	3.6, 15.1	2.8	−3.1, 9.3	18.4	9.7, 27.0
Age 18–59 years								
All years	0.3	0.0, 0.6	0.2	−0.2, 0.6	0.2	−0.3, 0.6	0.6	0.0, 1.2
2005–2006	0.1	0.0, 0.3	−0.5	−1.1, 0.1	0.1	−0.6, 0.6	−0.4	−1.2, 0.4
2006–2007	0.2	−0.3, 0.6	0.3	0.0, 0.7	−0.1	−1.0, 0.3	0.4	−0.5, 1.2
2007–2008	0.4	−0.1, 1.1	0.3	−0.4, 1.1	0.3	−0.5, 1.0	1.0	0.1, 1.9
2008–2009	0.6	0.0, 1.3	0.6	0.0, 1.3	0.4	−0.3, 1.4	1.5	0.5, 2.6
Age ≤17 years								
All years	−0.1	−0.3, 0.2	−0.1	−0.4, 0.3	−0.2	−0.7, 0.2	−0.3	−0.9, 0.2
2005–2006	0.0	−0.1, 0.1	0.0	−0.5, 0.5	−0.5	−1.1, 0.0	−0.5	−1.2, 0.16
2006–2007	−0.2	−0.6, 0.2	0.0	−0.3, 0.2	−0.2	−0.8, 0.3	−0.4	−1.1, 0.3
2007–2008	0.0	−0.5, 0.4	−0.1	−0.7, 0.4	−0.7	−1.3, 0	−0.9	−1.6, −0.2
2008–2009	0.0	−0.4, 0.5	0.0	−0.5, 0.5	0.4	−0.5, 1.4	0.4	−0.5, 1.4

Abbreviation: CrI, credible interval.

^a^ Estimates were obtained using the model adjusting for meteorological data allowing for annual variation in the association between type-specific influenza activity measures and mortality using a random-effects model.

Despite the strong seasonal pattern seen in deaths attributed to circulatory causes (Figure 3), the model estimated that influenza made little or no contribution to mortality in this category (probability of positive association, 46.9%). Unplanned analysis of deaths attributed to ischemic heart disease and cerebrovascular disease (subgroups of circulatory disease) (Web Figure 6) showed a similar lack of association between influenza and mortality (Web Table 11). There was, however, some evidence that influenza A was associated with increased mortality for these categories, while influenza B offered protection, with the net influenza association close to zero.

## DISCUSSION

On average, 6 people in every 100,000 were estimated to die each year in Thailand as a result of seasonal influenza, representing 4,000 deaths per year. There was no evidence of a net influenza contribution to circulatory disease deaths.

Strengths of our study include high-quality surveillance data, analytical methods that build on important recent methodological developments, and thorough sensitivity analyses (2, 12, 13). The credibility of our findings is strengthened by the lack of association between influenza and mortality in the 2 control groups. Although results were generally stable under different model assumptions, estimates of mortality associated with influenza B were close to zero in constant variance models. This might reflect difficulty in identifying contributions to mortality for a pathogen that circulates year round, as is the case for influenza B in Thailand (Figure 1).

Limitations include those of any observational study. Associations between influenza activity and mortality do not necessarily imply a simple chain of causation from infection to death. Additional limitations include the lack of spatial data and information on pathogens other than influenza. Also, because of changes in health care–seeking behavior for influenza-like illness following the 2009 pandemic, it was not possible to estimate mortality associated with the pandemic using the same approach. Accounting for these factors represents an important area for future research. In particular, it will be instructive to see if latitudinal variations in influenza-associated mortality reported elsewhere are also evident in Thailand (21).

Thailand is a developing middle-income tropical country with a predominantly rural population and a nominal per capita gross domestic product close to the world median (22, 23). There is no routine vaccination against *S. pneumoniae* and, in 2008, influenza vaccine sales amounted to only 1.6 per 100 people (24). No national estimates of total mortality caused by seasonal influenza A and B infections are available from any other developing country in the tropics. Our findings may therefore provide an important basis for generalization about the probable burden of seasonal influenza mortality outside high-income countries. Generalizations should, however, be made with caution. In South Africa (which has subtropical and temperate climate zones), influenza has been estimated via a Serfling-type model to cause 340 annual deaths per 100,000 population in those aged over 65 years, 5 times the corresponding rate in the elderly population in Thailand (10). Reasons for this large difference are not clear and need further investigation, but interactions with other pathogens may be one contributory factor (25).

The only previous comparable estimates in a tropical climate zone are from Singapore, a small, completely urbanized, and very high-income country. Using 2004–2006 data, influenza was estimated to account for a mean of 8.3 annual deaths per 100,000 population (5). Also in the tropics, a study in Bangladesh in 2009 (combining seasonal and pandemic influenza) estimated influenza-related mortality to be 11 per 100,000 (26). However, only deaths with influenza-like illness were considered, and influenza-like illness was ascertained retrospectively by interviewing household members the following year.

Seasonal influenza mortality has also been estimated in a number of cities in China with a subtropical climate. In Hong Kong and Guangzhou (both relatively developed populations with gross domestic product per capita 7 and 3 times higher than that of Thailand), estimates were 11.1 and 10.6 per 100,000 population, respectively (4); another study estimated influenza-associated mortality in 5 subtropical Chinese cities to be 11.3 per 100,000 (27). In the United States, annual influenza-related mortality was recently estimated to be 11.9 per 100,000 (2). These results suggest that Thailand experiences a similar or slightly lower influenza-related mortality than these much higher income populations. However, influenza-related mortality is highly age dependent and will be strongly affected by a population's age distribution. For example, the proportion of the population aged >60 years in Hong Kong is 19% (http://www.census2011.gov.hk/en/) but averaged only 11% in Thailand during 2005–2009. Adjusting for differences in age structure and calculating expected mortality for a world-standard age structure give a more informative comparison (28). This gives point estimates for standardized mortality that are very similar in Thailand and Hong Kong (8.8 vs. 8.5 per 100,000 population) (4).

Our findings diverged from those of previous studies in the relative importance of different influenza types. In the United States, influenza A(H1N1) has been estimated to make a small or negative contribution to mortality, while influenza B accounted for about one-fifth of influenza-related deaths (2). In Hong Kong, influenza B and A(H1N1) are estimated to account for about one-third and one-quarter as many deaths as A(H3N2) (4), while another study estimated that types A and B made similar contributions to mortality in northern (temperate) Chinese cities, while in southern (subtropical) cities, type B dominated (27). In contrast, we estimated that A(H1N1) made the largest contribution to mortality, and that type B was associated with roughly half the mortality associated with type A. Some of these differences may reflect chance variation associated with different virus types over the study periods. For example, A(H3N2) mortality appears to have declined following the emergence of the Fujian strain in 2003 (predating our data) (2).

A striking finding was the lack of association of seasonal influenza with circulatory disease mortality. This contrasts with high-income temperate and subtropical settings where influenza is estimated to make a major contribution to circulatory disease hospitalization and death (2, 29, 30). There are several possible explanations. First, “noisy” influenza data or inaccurate ICD-10 coding may prevent a true association from being found. However, although there is clearly potential for improvement in ICD-10 data from Thailand (as elsewhere) (31), the similar seasonal patterns for ischemic heart disease and cerebrovascular disease (Web Figure 6) and the associations between influenza and other ICD-10 codes previously linked with influenza mortality suggest that this explanation is unlikely. Second, because of differences in humidity, aerosol transmission of influenza may be much lower in the tropics than in temperature regions (32). There is evidence that droplet or contact-based spread of influenza is less likely to lead to typical influenza symptoms than is aerosol transmission, and this could conceivably lead to differences in cause-specific mortality (33). Third, associations reported elsewhere between influenza activity and circulatory disease deaths could be mediated by another factor, such as secondary bacterial infections, and such interactions could differ in tropical and temperate zones (34). Fourth, the greater importance of acute rather than chronic circulatory disease in Thailand might be responsible for the different associations with influenza. Finally, it is possible that the high incidence and year-round circulation of influenza B result in more frequent but milder infections that protect against other more severe infections through nonspecific immunity. Interestingly, in Brazil, the 2009 pandemic was associated with a very large increase in respiratory mortality but no association with circulatory mortality (35).

In summary, we have shown a substantial but previously hidden mortality burden due to influenza in a tropical middle-income country, less than 2% of which is likely to be accounted for by hospitalized cases of influenza pneumonia (36).

## Supplementary Material

Web Material
