# Supplementary material for: Mortality Attributable to Seasonal Influenza A and B Infections in Thailand, 2005–2009: A Longitudinal Study
Source: Am J Epidemiol. 2015 Apr 20;181(11):898–907. doi: 10.1093/aje/kwu360 (PMC4445392; doi:10.1093/aje/kwu360)
Supplement: Web Material [file supp_181_11_898__index.html]

Mortality Attributable to Seasonal Influenza A and B Infections in Thailand, 2005–2009: A Longitudinal Study — Web Material 

# Mortality Attributable to Seasonal Influenza A and B Infections in Thailand, 2005–2009: A Longitudinal Study

## Web Material

Web Material

**Files in this Data Supplement:**

- Web Material - Pdf file
