# Supplementary material for: Mortality Attributable to Seasonal Influenza A and B Infections in Thailand, 2005–2009: A Longitudinal Study
Source: Am J Epidemiol. 2015 Apr 20;181(11):898–907. doi: 10.1093/aje/kwu360 (PMC4445392; doi:10.1093/aje/kwu360)
Supplement: Web Material [file supp_kwu360_kwu360supp.pdf]

Web Appendix.

Model details.

We defined an influenza activity measure, $B_{j,i}$, for each of the three influenza types (A/H1N1, A/H3N2, and B). This was given as the product of the proportion of laboratory tests positive for influenza type *j* in week *i* ($P_{j,i}$) and the total number of patients with influenza like illness (ILI) in week *i* (I_i). The number of patients with ILI due to causes other than influenza may vary over time, as may the probability that laboratory confirmation for influenza is sought in a patient seeking medical attention with ILI (T_i). The value of $B_{j,i}$ will not be affected by such variation and will be proportional to the number of true cases with influenza type *j* in week *i*, ($C_{j,i}$), provided that the probability that a patient with an influenza type *j* infection seeks medical attention with ILI (M_j), and the test sensitivity (*s*), do not vary with time. This follows since $P_{j,t}= C_{j,i} \times M_j \times T_i \times s/(I_i \times T_i)$. Rearranging gives
$B_{j,i} = C_{j,i} \times M \times s.$

We also define the following variables:

D_i represents deaths for week *i*
w_i represents the number of weeks since the first observation
W_i represents the week number of the year (1..53)
R_i represents standardized weekly rainfall
H_i represents standardized weekly relative humidity
M_i represents standardized weekly maximum temperature
f_A and f_B represent the proportional contribution to deaths of influenza A and B cases one week previously

The full model with non-linear adjustment for meteorological data, a random-effects model for influenza coefficients and first order autoregressive conditional heteroscedastic (ARCH) process for the variance is defined below:

$D_i \sim \text{Normal}(\mu_i, \sigma_i^2)$

$$\mu_i = a + b_{\text{year}_i}\, H1N1proxy_i + c_{\text{year}_i}\, H3N2proxy_i + d_{\text{year}_i}\, Bproxy_i + \beta_1 w_i + \beta_2 w_i^2 + \Sigma_{j=1..K_1} s_j\, z_{1ij} + \Sigma_{j=1..K_2} r_j\, z_{2ij} + \Sigma_{j=1..K_3} h_j\, z_{3ij} + \Sigma_{j=1..K_4} m_j\, z_{4ij} + \theta_i$$

$e_t = D_i - \mu_i$
$\sigma_i^2 = \alpha_0 + \alpha_1 e^2_{t-1}$

$H1N1proxy_i = f_A\, B_{1,i-1} + (1-f_A)\, B_{1,i-2}$
$H3N2proxy_i = f_A\, B_{2,i-1} + (1-f_A)\, B_{2,i-2}$
$Bproxy_i = f_B\, B_{3,i-1} + (1-f_B)\, B_{3,i-2}$

$b_k \sim \text{Normal}(\mu_b, \sigma_b^2)$
$c_k \sim \text{Normal}(\mu_c, \sigma_c^2)$
$d_k \sim \text{Normal}(\mu_d, \sigma_d^2)$
$s_j \sim \text{Normal}(0, \sigma_s^2)$
$r_j \sim \text{Normal}(0, \sigma_r^2)$
$h_j \sim \text{Normal}(0, \sigma_h^2)$
$m_j \sim \text{Normal}(0, \sigma_m^2)$

$z_{1ij} = \max[0,(W_i - \kappa_{1j})^3]$
$z_{2ij} = \max[0,(R_i - \kappa_{2j})^3]$
$z_{3ij} = \max[0,(H_i - \kappa_{3j})^3]$
$z_{4ij} = \max[0,(M_i - \kappa_{4j})^3]$

Where the κ_{ij} terms correspond to the spline “knots”. For $\kappa_{1.}$ (used to model background seasonal variation) these were placed at four week intervals, starting in week 2. The other knots were by default placed at -1,0 and 1.

The second order random walk prior is defined by

$\theta_i | \theta_{-i} \sim \text{Normal}(2\theta_{i+1} - \theta_{i+2}, \phi)$ for i =1
$\sim \text{Normal}((2\theta_{i-1} + 4\theta_{i+1} - \theta_{i+2})/5 , \phi/5)$ for i =2
$\sim \text{Normal}((-\theta_{i-2} + 4\theta_{i-1} + 4\theta_{i+1} - \theta_{i+2})/6 , \phi/6)$ for i =3...N-2
$\sim \text{Normal}((-\theta_{i-2} + 4\theta_{i-1} + 2\theta_{i+1})/5 , \phi/5)$ for i =N-1
$\sim \text{Normal}(-\theta_{i-2} + 2\theta_{i-1}, \phi)$ for i =N

Priors for μ_b , μ_c and μ_d: ~Normal(0,10^6).
Priors for f_A and f_B: ~Beta(1,1).

Prior distributions for the variance terms were specified through the precision (the reciprocal of the variance) which was given as a Gamma distribution with shape 0.001 and scale 1000 (corresponding to a mean of 1 and a variance of 1000).

All model posteriors reported are based on summaries of at least 10 million Markov chain Monte Carlo iterations following convergence. Convergence was assessed by visual inspection of the chains and by using the Gelman-Rubin diagnostic[1].

Estimates of numbers of influenza-related deaths using the above family of models were converted to mortality rates using year-specific estimates of the population size in each age group. We used data from the 2000 census in Thailand as a starting point to derive numbers in each age group in each year (http://web.nso.go.th/), then adjusted these using World Bank estimates for the annual percentage changes in the proportion of the population aged >=65 years and <15 years between 2000 and 2009, assuming the proportional changes to be the same in those aged >=60 and <18 years respectively. For the total population size each year we also used World Bank estimates.

Web Table 1. Comparison of model fit for 12 different generalized additive models. Model fitting used penalized likelihood maximization using the *mgcv* package in *R*. All models considered included covariates *H1N1proxy*, *H2N2proxy* and *Bproxy*, used a p-spline with 12 knots to model seasonal variation, and chose values of f_A and f_B to maximise the R^2 of the fit of model 8 below under the constraint that they were equal to each other.

Model	AIC
a. Gaussian. No long-term trends.	2828.5
b. Gaussian. Long-term trends modelled using a linear term for year.	2826.8
c. Gaussian. Long-term trends modelled using a quadratic term for year.	2826.8
d. Gaussian. Long-term trends modelled using a cubic term for year.	2947.6
e. Gaussian. Long-term trends modelled using a linear term for week number.	2826.8
f. Gaussian. Long-term trends modelled using a quadratic term for week number.	2824.6
g. Gaussian. Long-term trends modelled using a cubic term for week number.	2825.0
h. Gaussian. Long-term trends modelled using a thin plate regression spline.	2824.5
i. Gaussian. Long-term trends modelled using a p-spline.	2824.2
j. Poisson with identity link. No long-term trends.	4035.9
k. Poisson with identity link. Long-term trends modelled using a quadratic term for week number.	3965.9
l. Quasipoisson with identity link. Long-term trends modelled using a p-spline.	3895.6

Web Table 2. Comparison of model fit for seven different models. Two measures of model fit which have been proposed are shown: E(*D*)+ p_v and E(*D*) + p_d (also referred to as the Deviance Information Criterion)[3,4]. Here p_v is defined as half the variance of the posterior deviance, *D*, and p_d is defined as the posterior mean deviance minus the deviance of the posterior means. In both cases lower values of the statistic indicate better fit.

Model	E(D)+ p_v	E(D)+ p_d
1. No adjustment for meteorological data; time-invariant influenza coefficients (β_j)	2834	2829
2. Linear adjustment for meteorological data; time-invariant influenza coefficients	2808	2805
3. Non-linear adjustment for meteorological data; time-invariant influenza coefficients	2815	2799
4. No adjustment for meteorological data; year-specific influenza coefficients	2831	2823
5. No adjustment for meteorological data; random-effects model for influenza coefficients	2834	2824
6. Non-linear adjustment for meteorological data; random-effects model for influenza coefficients	2807	2786
7. Non-linear adjustment for meteorological data; year-specific influenza coefficients	2809	2793

Web Table 3: Estimated all-cause annual mortality due to seasonal influenza in Thailand, using Model 1 (no adjustment for meteorological data, and assuming no annual variation in the association between influenza proxy measures and mortality).

	Estimated yearly mortality per 100,000 (95% CrI)		
Year	H1N1	H3N2	B
2005-6	0.4 (0.1, 0.7)	4.8 (0.2, 9.5)	-2.6 (-6.9, 1.7)
2006-7	5.3 (1.7, 8.7)	1.5 (0.1, 2.9)	-1.1 (-3.1, 0.8)
2007-8	2.5 (0.8, 4.1)	2.5 (0.1, 4.9)	-3.7 (-9.8, 2.5)
2008-9	5.4 (1.8, 8.9)	3.1 (0.1, 6.0)	-2.6 (-7.0, 1.8)
Combined years	3.4 (1.1, 5.7)	3.0 (0.1, 5.8)	-2.5 (-6.7, 1.7)

Web Table 4: Estimated all-cause annual mortality due to seasonal influenza in Thailand, using Model 2 (linear adjustment for meteorological data, and assuming no annual variation in the association between influenza proxy measures and mortality).

	Estimated yearly mortality per 100,000 (95% CrI)		
Year	H1N1	H3N2	B
2005-6	0.3 (0.0, 0.6)	3.4 (-0.9, 7.8)	-2.7 (-6.7, 1.4)
2006-7	3.9 (0.6, 7.2)	1.0 (-0.3, 2.4)	-1.2 (-3.0, 0.7)
2007-8	1.9 (0.3, 3.5)	1.8 (-0.5, 4.0)	-3.8 (-9.6, 2.0)
2008-9	3.9 (0.6, 7.3)	2.3 (-0.6, 5.0)	-2.6 (-6.8, 1.4)
Combined years	2.5 (0.4, 4.6)	2.2 (-0.6, 4.8)	-2.5 (-6.5, 1.3)

Web Table 5: Estimated all-cause annual mortality due to seasonal influenza in Thailand, using Model 3 (non-linear adjustment for meteorological data, and assuming no annual variation in the association between influenza proxy measures and mortality).

	Estimated yearly mortality per 100,000 (95% CrI)		
Year	H1N1	H3N2	B
2005-6	0.4 (0.2, 0.7)	4.6 (0.2, 8.9)	-2.6 (-6.6, 1.6)
2006-7	5.2 (1.9, 8.5)	1.4 (0.1, 2.7)	-1.1 (-3.0, 0.8)
2007-8	2.4 (0.9, 4.0)	2.4 (0.1, 4.6)	-3.6 (-9.4, 2.4)
2008-9	5.4 (2.0, 8.7)	2.9 (0.1, 5.7)	-2.5 (-6.7, 1.7)
Combined years	3.4 (1.2, 5.5)	2.8 (0.1, 5.5)	-2.5 (-6.4, 1.6)

Web Table 6: Estimated all-cause annual mortality due to seasonal influenza in Thailand, using Model 4 (no adjustment for meteorological data, but allowing year-specific estimates for the association between influenza proxy measures and mortality).

	Estimated yearly mortality per 100,000 (95% CrI)		
Year	H1N1	H3N2	B
2005-6	0.7 (-2.6, 4.0)	-3.1 (-9.2, 3.0)	6.8 (-0.6, 14.4)
2006-7	4.7 (2.6, 19.9)	7.8 (3.0, 12.5)	5.1 (-1.6, 15.7)
2007-8	14.9 (4.5, 23.8)	3.2 (-7.1, 13.5)	-9.9 (-19.6,-0.2)
2008-9	8.2 (2.0, 14.3)	6.9 (-1.4, 15.1)	-10.9 (-23.6, 1.7)
Combined years	7.1 (3.8, 10.5)	3.7 (-0.6, 7.9)	-2.4 (-8.3, 4.0)

Web Table 7: Estimated all-cause annual mortality due to seasonal influenza in Thailand, using Model 5 (no adjustment for meteorological data, but allowing for annual variation in the association between influenza proxy measures and mortality using a random effects model).

	Estimated yearly mortality per 100,000 (95% CrI)		
Year	H1N1	H3N2	B
2005-6	0.6 (-0.3, 1.8)	-0.5 (-6.7, 6.0)	1.6 (-4.9, 9.8)
2006-7	5.5 (1.0, 9.7)	6.7 (1.6, 11.4)	0.5 (-4.2, 7.8)
2007-8	4.4 (0.9, 13.8)	4.0 (-3.8, 11.7)	-3.5 (-12.6, 4.5)
2008-9	6.1 (1.5, 11.0)	5.8 (-1.2, 13.2)	-4.8 (-17.5, 3.4)
Combined years	4.1 (1.4, 7.4)	4.0 (0.2, 7.8)	-1.5 (-6.8, 3.5)

Web Table 8: Estimated all-cause annual mortality due to seasonal influenza in Thailand, using Model 6 (non-linear adjustment for meteorological data, allowing allowing for annual variation in the association between influenza proxy measures and mortality using a random effects model).

	Estimated yearly mortality per 100,000 (95% CrI)		
Year	H1N1	H3N2	B
2005-6	0.6 (-0.1, 1.9)	-0.6 (-6.1, 5.0)	3.9 (-2.7, 12.0)
2006-7	5.6 (1.6, 9.5)	7.7 (3.2, 12.0)	1.0 (-4.0, 8.1)
2007-8	4.2 (0.9, 12.7)	1.8 (-5.6, 9.0)	-1.4 (-9.7, 5.9)
2008-9	5.7 (1.4, 10.2)	5.0 (-1.8, 11.9)	-4.3 (-16.8, 4.2)
Combined years	4.1 (1.4, 7.2)	3.5 (0.0, 7.0)	-0.2 (-5.0, 4.5)

Web Table 9: Estimated all-cause annual mortality due to seasonal influenza in Thailand, using Model 7 (non-linear adjustment for meteorological data, allowing year-specific estimates for the association between influenza proxy measures and mortality).

	Estimated yearly mortality per 100,000 (95% CrI)		
Year	H1N1	H3N2	B
2005-6	0.9 (-2.1, 4.1)	2.2 (-2.3, 6.7)	3.0 (-3.7, 10.0)
2006-7	4.5 (-0.3, 9.3)	6.9 (2.5, 11.4)	-3.4 (-12.5, 5.7)
2007-8	13.7 (5.3, 22.2)	-3.1 (-11.9, 5.6)	-9.6 (-18.6, -0.5)
2008-9	10.4 (4.9, 16.1)	10.3 (2.8, 17.8)	-5.5 (-16.2, 4.9)
Combined years	7.4 (4.1, 10.7)	4.1 (0.2, 8.0)	-3.9 (-9.4, 1.7)

Web Table 10: Regression coefficients relating influenza activity to all cause mortality using model 6. Results given are posterior means (and 95% CrIs).

	Estimated yearly mortality per 100,000 (95% CrI)		
Year	H1N1	H3N2	B
2005-6	3.1 (-0.3, 10.1)	-0.1 (-1.8, 1.6)	2.0 (-1.4, 6.2)
2006-7	2.5 (0.8, 4.2)	7.3 (3.0, 11.2)	1.2 (-4.7, 9.2)
2007-8	3.8 (0.8, 11.4)	1.1 (-3.2, 5.2)	-0.7 (-3.7, 2.0)
2008-9	2.4 (0.6, 4.3)	2.3 (-0.8, 5.6)	-2.4 (-8.9, 2.0)

Web Table 11.Estimated Mortality Attributed to Influenza for Deaths With ICD-10 Codes Corresponding to Circulatory Disease, Ischemic Heart Disease and Cerebrovascular Disease in Thailand, 2005-2009. Estimates are from model 5 (unadjusted) and model 6 under the assumptions of constant variance.

	A/H1N1		A/H3N2		B		Total influenza A		Total influenza A & B	
Recorded cause of death	mean	95% CrI	mean	95% CrI	mean	95% CrI	mean	95% CrI	mean	95% CrI
Circulatory	0.5	-0.1, 1.2	0.3	-0.3, 1.0	-0.7	-1.9, 0.4	0.8	-0.1, 1.9	0.1	-1.2, 1.4
Ischemic heart disease	0.5	0.2. 0.7	0.0	-0.3, 0.3	-0.5	-0.9, -0.2	0.5	0.1, 0.9	0.0	-0.5, 0.4
Cerebrovascular disease	0.3	0.0, 0.6	0.2	-0.1, 0.5	-0.2	-0.7, 0.3	0.5	0.1, 1.0	0.3	-0.3, 0.9
Circulatory (unadjusted)	0.7	0.1, 1.3	0.3	-0.4, 1.0	-1.0	-2.2, 0.2	0.9	0.0, 2.0	0.0	-1.4, 1.3
Ischemic heart disease (unadjusted)	0.2	-0.3, 0.7	0.2	-0.4, 0.7	-0.4	-1.1, 0.3	0.4	-0.3, 1.1	0.0	-1.0, 0.9
Cerebrovascular disease (unadjusted)	0.4	0.1, 0.7	0.2	-0.1, 0.4	-0.2	-0.7, 0.2	0.6	0.1, 1.0	0.4	-0.2, 0.9

Web Figure 1. Samples from the posterior distributions of estimated mortality and model deviance. Results are from model 6 from Web Table 1 applied to all cause (and all age) mortality data. Each graph shows 10,000 samples, where every 1000th iteration is sampled following convergence.

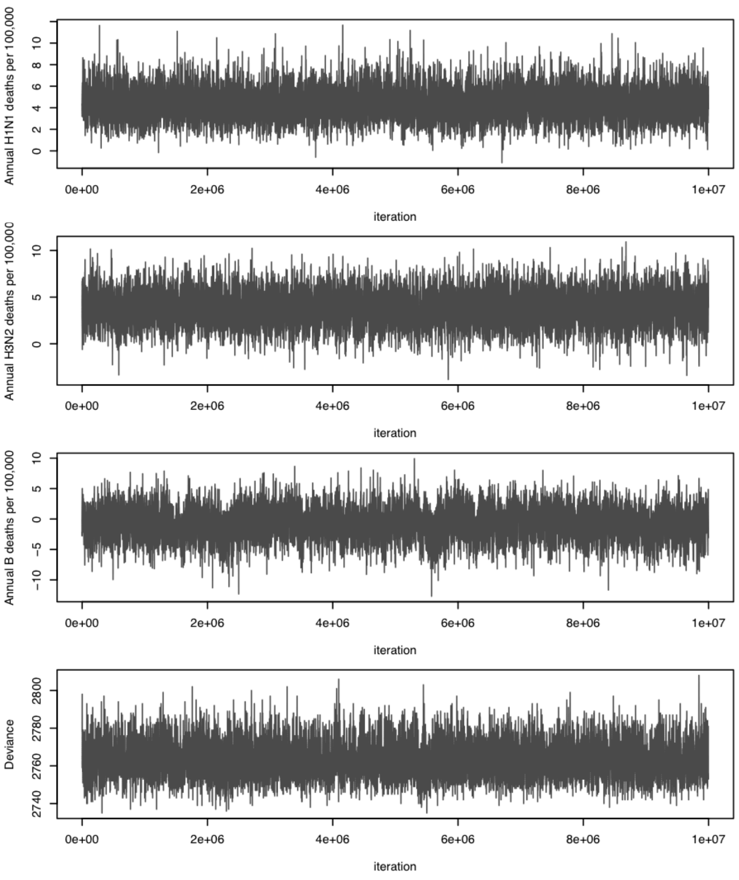

Web Figure 2. Estimated seasonal variation accounted for by the periodic P-spline term. The graph shows the posterior mean (solid line) and associated 95% and 80% credible intervals (light and dark shading) for the difference in mortality by week number using model 6 applied to all cause mortality data.

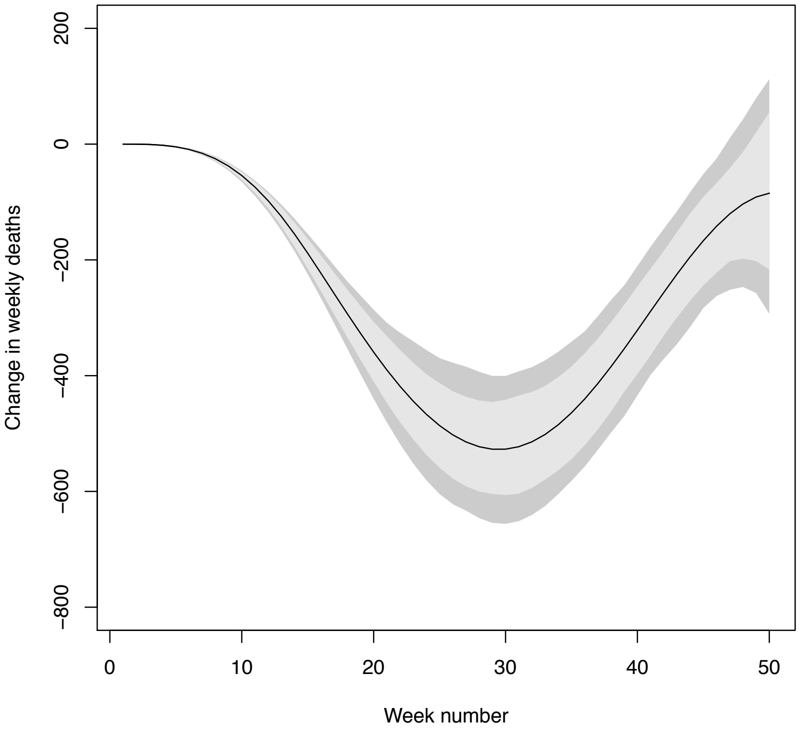

Web Figure 3. Relationship between rainfall, relative humidity, temperature and changes in all cause mortality. Results are based on model 6 estimates after adjusting for regular seasonal variation using a periodic P-spline. The top row shows data obtained from monitoring stations in Bangkok. The bottom two rows show changes in the expected number of weekly deaths for a given value of the meteorological variable (solid line) and associated 95% and 80% credible intervals (light and dark shading). P-splines are used to model the non-linear relationship between the meteorological data and mortality; results are shown for model 6 with three knots (middle row) at z-scores of -1,0,1, and for a model with 5 knots (bottom row) at z-scores of -3,-1,0,1, 3.

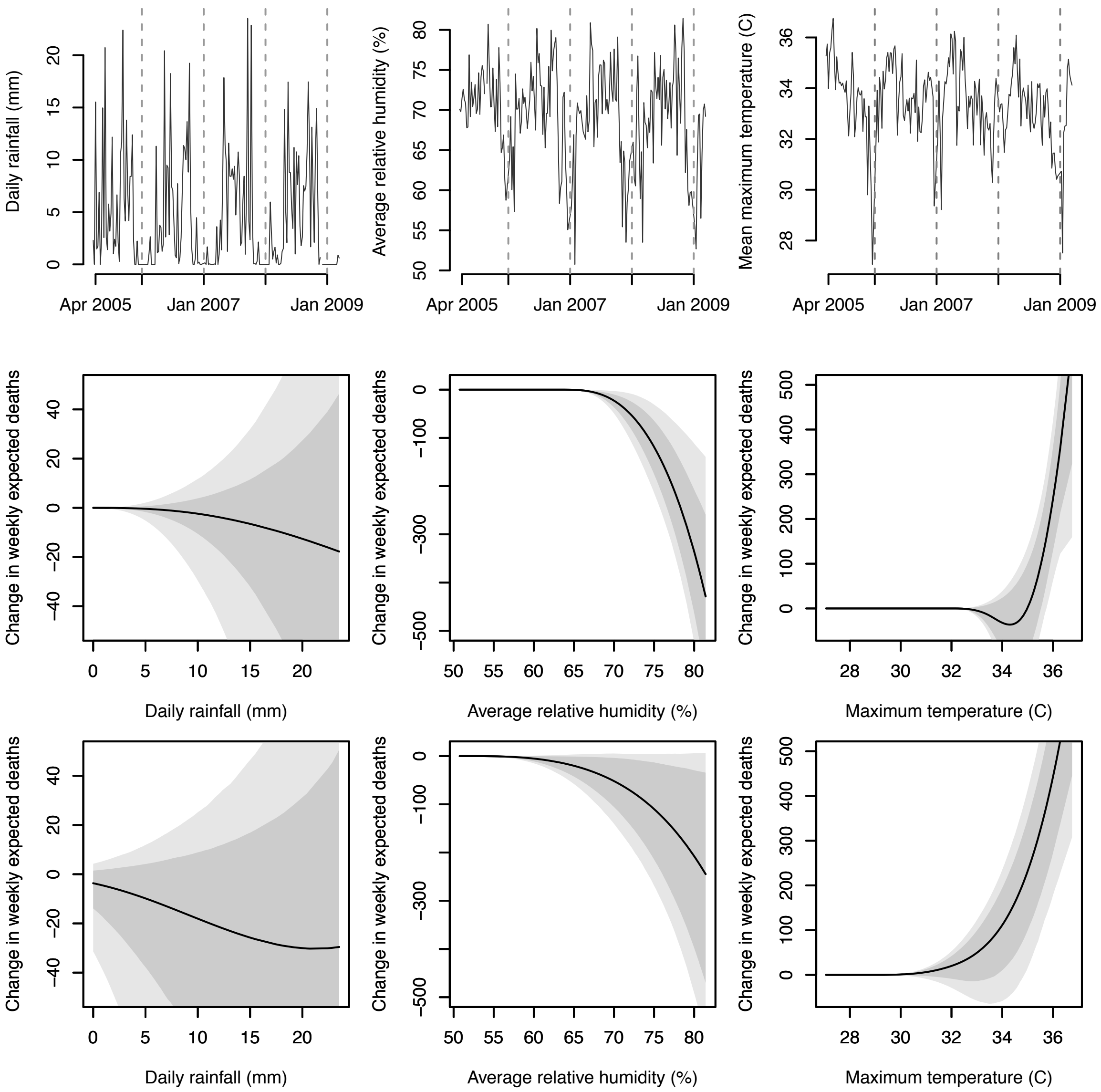

Web Figure 4. Annual variation in the relationship between influenza activity and associated mortality. Plots show posterior densities of the regression coefficients, β_j, relating the measure of activity with influenza type *j* to all cause mortality. Results are from model 6 (adjusting for meteorological data, and allowing for annual variation in the association between influenza activity measures and mortality using a random effects model). Posterior medians and group means are shown by broken and solid vertical vertical lines respectively.

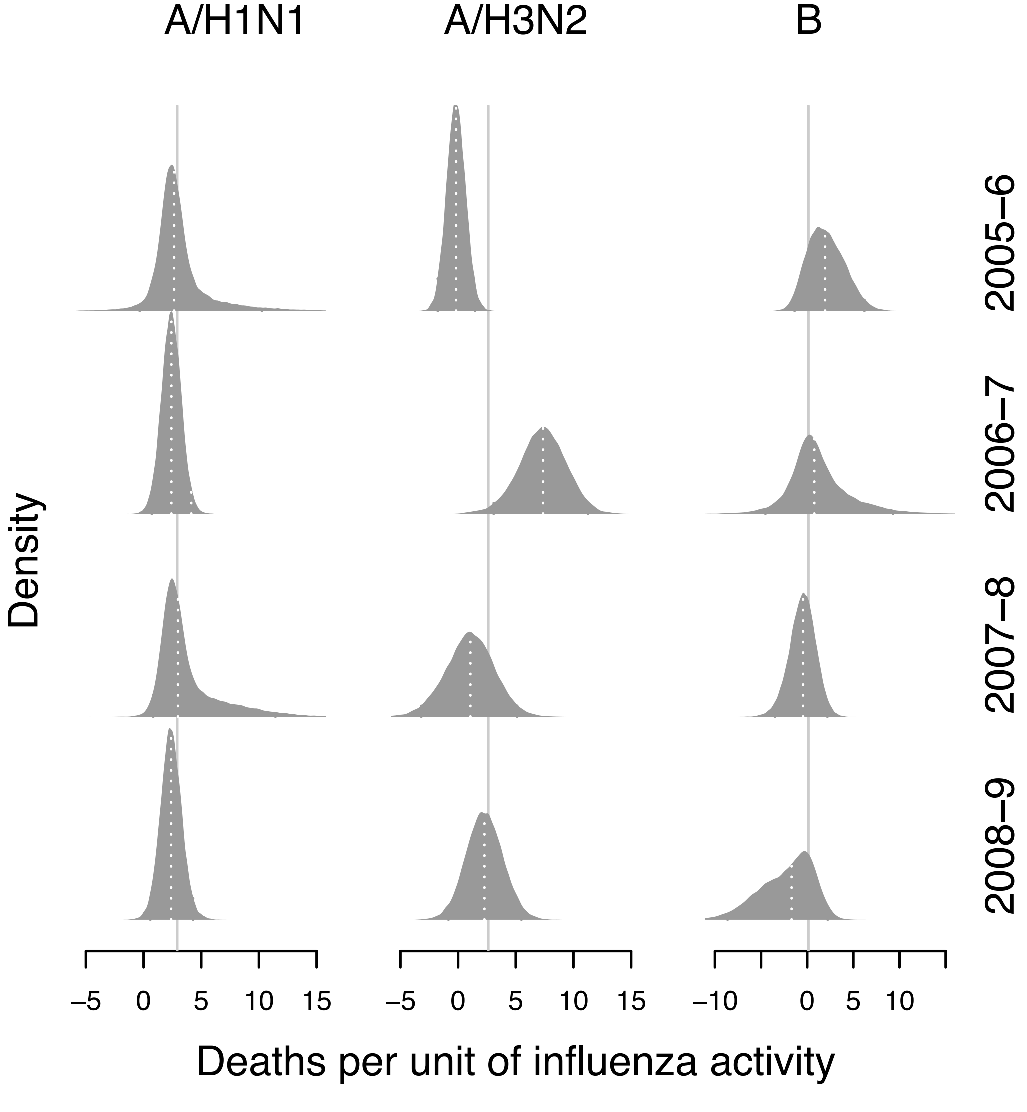

Web Figure 5. Correlations between posterior distributions for the regression coefficients relating influenza proxies to all cause mortality. Number in the top right corner give Kendall's correlation coefficient. Results are from model 6 applied to all cause (and all age) mortality data.

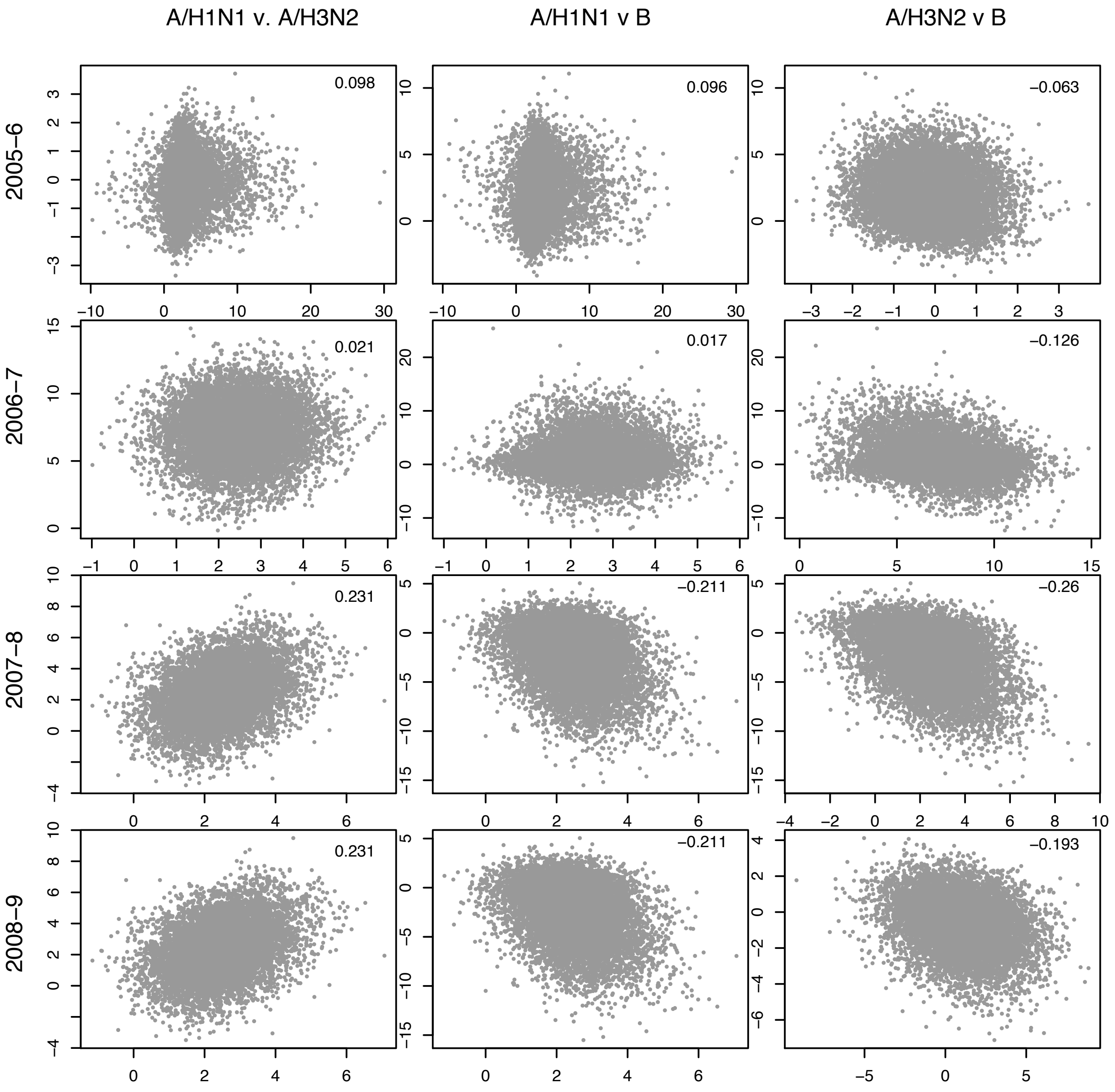

Web Figure 6. Deaths due to all circulatory disease, ischemic heart disease, and cerebrovascular disease in Thailand. Corresponding ICD-10 codes are I00-I99 (all circulatory disease), I20-I25 (ischemic heart disease), and I60-I69 (cerebrovascular disease).

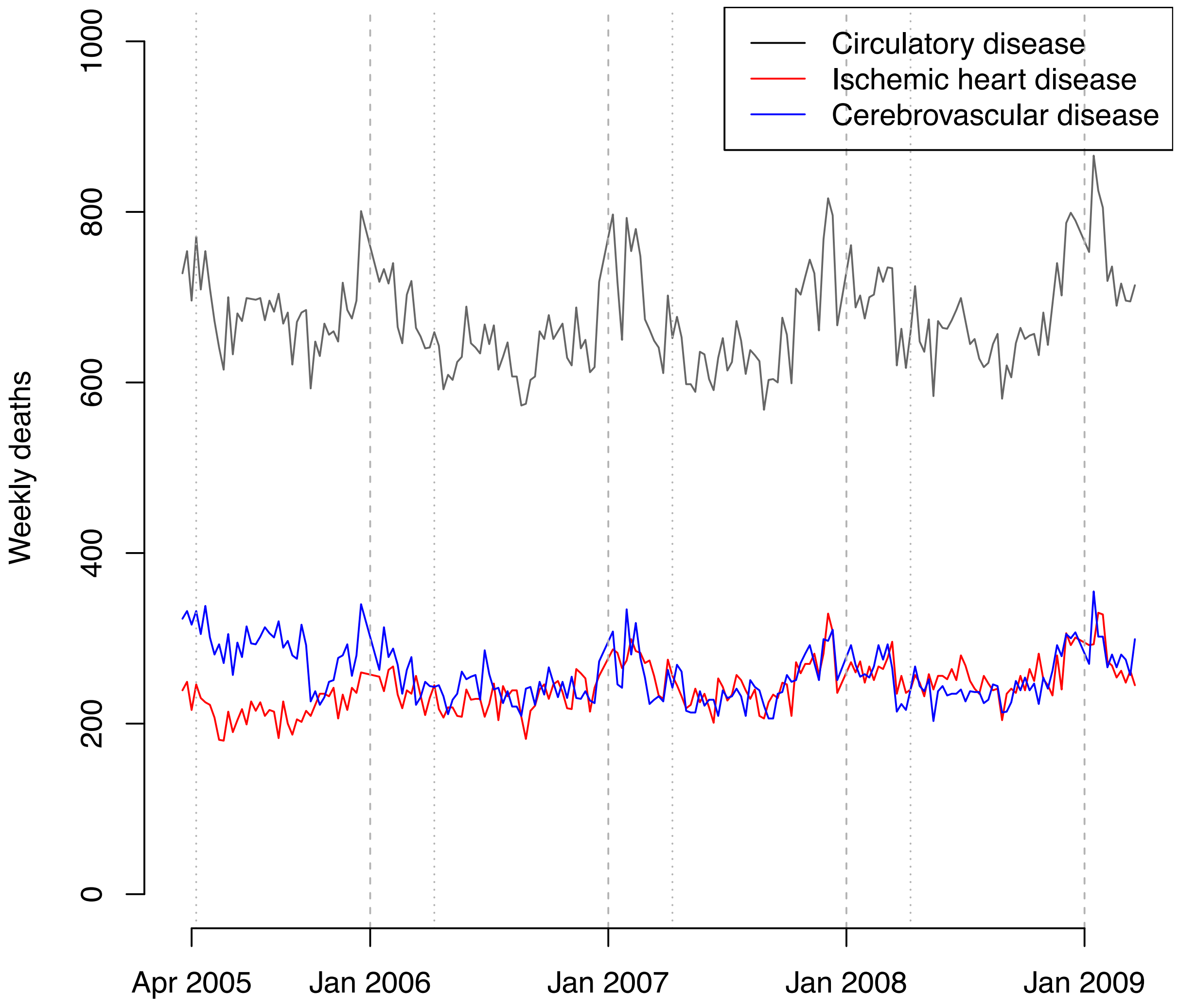

References

1 Gelman, A and Rubin, DB (1992) Inference from iterative simulation using multiple sequences, Statistical Science 7: 457-511.

2 Akaike H. A new look at the statistical model identification (1974) IEEE Transactions on Automatic Control 19: 716–723.

3 Gelman A, Carlin JB, Stern HS, Rubin DB (2004) Bayesian Data Analysis, Boca Raton: Chapman & Hall/CRC. pp. 157-196.

4 Spiegelhalter DJ, Best NG, Carlin BP, van der Linde, A (2002) Bayesian measures of model complexity and fit. J R Stat Soc B 64: 583–616.
